# Identification of RNA Modification-Associated Alternative Splicing Signature as an Independent Factor in Head and Neck Squamous Cell Carcinoma

**DOI:** 10.1155/2022/8976179

**Published:** 2022-09-13

**Authors:** Jie Liu, Wenmin Deng, Zhiwen Xiao, Xiaofeng Huang, Minmin Lin, Zhen Long

**Affiliations:** Department of Otorhinolaryngology Head and Neck Surgery, The Sixth Affiliated Hospital, Sun Yat-sen University, Guangzhou, China

## Abstract

**Objective:**

Head and neck squamous cell carcinoma (HNSCC) is a highly heterotopic malignant tumor. Alternative splicing (AS) and RNA modification have been reported to be involved in tumorigenesis. Therefore, we constructed RNA modification-associated AS (RMA-AS) signature model to predict the prognosis of HNSCC.

**Methods:**

AS events and RNA-modified gene expression information were downloaded from TCGA-HNSCC database. Univariate Cox regression analysis was employed for analyzing prognosis-related AS events. RMA-AS events were obtained by constructing a coexpression network between RNA modification-associated genes and AS events using WGCNA package. The prognostic signatures were analyzed by LASSO, univariate Cox, and multivariate Cox regression. Kaplan-Meier survival analysis, proportional hazard model, and ROC curve were performed to verify the prognostic value. “ESTIMATE” R package, ssGSEA algorithm, and CIBERSORT method were used to detect immune microenvironment in HNSCC. Cytoscape was utilized to build a regulatory network of splicing factor-regulated RMA-AS.

**Results:**

There were 16,574 prognostic AS events and 4 differentially expressed RNA modification-associated genes in HNSCC. Based on RMA-AS events, we obtained a risk model consisting of 14 AS events, named RMA-AS_Score. The samples were divided into RMA-AS_Score high- and RMA-AS_Score low-risk groups, according to the risk score. The RMA-AS_Score high group was related to poor prognosis. Moreover, the RMA-AS_Score signature was an independent prognostic predictor and was related to tumor grade. Meanwhile, the AUC value of RMA-AS_Score was 0.652, which is better than other clinical characteristics. Besides, a nomogram prediction model of quantitative prognosis has also been developed, which has robust effectiveness in predicting prognosis. In addition, the prognostic signature was observably related to immune microenvironment and immune checkpoint. Finally, 14 splicing factors were identified and constructed into a network of splicing factor-regulated RMA-AS.

**Conclusion:**

We identified the RMA-AS signature of HNSCC. This signature could be treated as an independent prognostic predictor.

## 1. Introduction

Head and neck squamous cell carcinoma (HNSCC) is a heterogeneous malignant tumor with a high mortality rate [1], of which the survival rate of patients with advanced cancer is only 34.9% [2]. Although tumor, lymph node, and TNM categories are widely used prognostic tools in cancer with the progress of medical technology [3], they have little effect on accurately predicting the prognosis of HNSCC. Furthermore, since the high heterogeneity of HNSCC is a great challenge to the treatment effect, there is an urgent need for biomarkers to contribute to make an accurate early diagnosis of HNSCC in clinic, improve the prognosis, and provide reference for the development of individualized drugs.

Alternative splicing (AS) is an important modification mechanism in gene expression regulation [4]. Abnormal regulation of AS contributes to tumorigenesis and pathogenesis [5]. AS events can be utilized to predict the prognosis of cancer, which has gradually attracted human attention. For example, seven-AS event prognostic signature can independently predict the overall survival (OS) of uveal melanoma patients [6]. In colon cancer, systematic analysis of the relationship between AS events and tumor immune infiltration significantly improves the ability of prognosis prediction [7]. However, there are few studies on the prognosis of AS events in HNSCC.

Chemical modification is a highly specific and effective method to regulate the function of biological macromolecules, and the common modification methods are DNA and protein modification [8]. However, as science advances, RNA modification is a new frontier in this field. Human diseases are associated with the disorder of RNA modification, including cancer, cardiovascular diseases, and metabolic diseases [9]. M6A is a common RNA modification, and it has been proved that the change of m6A regulation gene is correlated with the occurrence and transfer of HNSCC [10]. In addition, tRNA N7-methylguanosine (m7G) modification also is involved in driving the progression of HNSCC. These evidences suggest that RNA modification is deeply involved in the occurrence and development of HNSCC. Considering that RNA modification-associated AS (RMA-AS) regulates mRNA attenuation and epigenetic changes exist in all human cancers, the identification of RMA-AS events in cancer has an important impact on the understanding of tumor pathogenesis and progression.

In this study, we utilized The Cancer Genome Atlas (TCGA) data to focus on AS events and abnormally expressed RNA modification-associated genes and correlated them with HNSCC clinicopathological and prognostic information to construct a prognostic model (RMA-AS_Score). Next, the clinical application value of RMA-AS_Score model in HNSCC was discussed by systematically analyzing the prognostic signatures of RMA-AS_Score. Then, the correlation between prognostic signature and immune microenvironment and immunotherapy was studied. Finally, a regulatory network of splicing factor-regulated RMA-AS was constructed to elucidate the underlying mechanisms involved in HNSCC development.

## 2. Materials and Methods

### 2.1. Data Download

**Figure 1:**
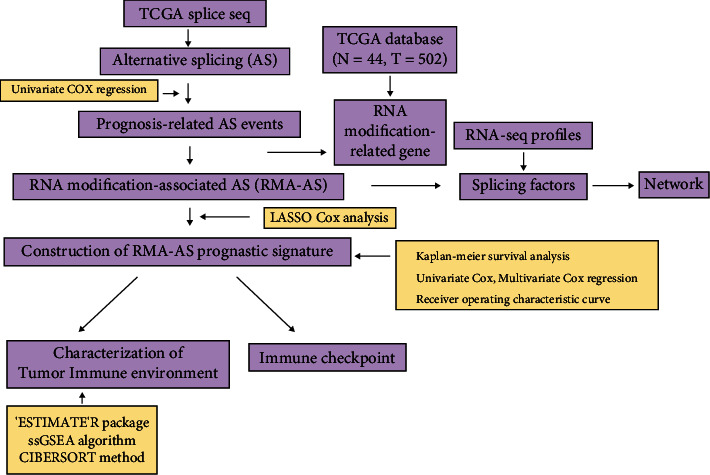
Flow chart of the comprehensive analysis process.

We first acquired the transcriptome and clinical information of the HNSCC patients (normal = 44 and tumor = 502) from TCGA-HNSCC database (https://portal.gdc.cancer.gov/) for subsequent analysis. The AS data were obtained from TCGA SpliceSeq (https://bioinformatics.mdanderson.org/TCGASpliceSeq/). The downloaded AS data contains the following seven types of AS events: alternate acceptors (AA), retained intron (RI), alternate donors (AD), alternate promoters (AP), exon skip (ES), alternate terminators (AT), and mutually exclusive exons (ME). Samples were screened according to the percent spliced in PSI value > 0.75 as the filtration cut-off point. The analysis flow chart is referred to Figure 1.

### 2.2. Approach of AS Event Identification

In TCGA SpliceSeq, the PSI value to quantify AS events was detected and then calculated. By using the UpSet R package, the UpSet plot showed the seven types of AS events. Subsequently, univariate Cox regression analysis was performed to detect the correlation between AS events and prognosis of HNSCC, which was also represented by the UpSet plot.

### 2.3. Expression Analysis of Genes Associated with RNA Modification

The expression of RNA modification-associated genes was normalized by Limma package, and the abnormally expressed genes in tumor and normal samples were screened by difference analysis, where the expression differences were characterized by |log_2_FC| ≥ 1 and FDR < 0.05.

### 2.4. Analysis of AS Associated with RNA Modification

We used the “WGCNA” (weighted gene coexpression network analysis) software package to correlate AS events with RNA modification-associated genes, referring to other reports [11]. Next, we performed Spearman's correlation and module eigengenes analysis of RNA modification-related gene expression association with AS events to acquire RMA-AS events.

### 2.5. Construction of Prognostic Signature

Firstly, we used the LASSO regression analysis for determining the candidate of each splicing mode to obtain a risk model consisting of 14 AS events (RMA-AS_Score). According to the risk score of each patient in different RMA-AS events, we divided the patients into high- and low-risk groups with the median as the critical value. Risk score = Σ(*α*_*i*_∗Exp_*i*_), where *α*_*i*_ means the weight of the each signature and Exp_*i*_ means the expression value. Also, we drew risk score distribution map, survival state map, and heatmap of PSI value to evaluate the RMA-AS_Score signature. The “survminer” package was used for Kaplan-Meier survival curve analysis (*P* < 0.05). In addition, univariate and multivariate Cox regression analyses were used to determine whether the RMA-AS_Score signature can be regarded as an independent factor for predicting the prognosis of HNSCC.

### 2.6. Validation of Prognostic Signature

We used stratified survival analysis to further verify the prognostic performance independent from clinical characteristics such as gender, tumor stage, and pathological grade. Next, we assessed the prognostic value of the signature by conducting the time-dependent receiver operating characteristic (ROC) curves (area under the curve, AUC > 0.6).

### 2.7. Construction of Prognostic Nomogram

To quantitatively predict the OS of HNSCC patients, we established a prognostic nomogram including as RMA-AS_Score model and other clinical variables to estimate the OS probability at 1, 2, and 3 years. Then, we drew the calibration curve of the nomogram with predictive value. The calibration curve is close to 45°, indicating that the model constructed has good prediction ability.

### 2.8. Correlation between Risk Score and Characteristics of Tumor-Infiltrating Immune Cells

Spearman's correlation was employed to analyze the correlation between tumor immune cell infiltration and prognostic risk score. Single sample gene set enrichment analysis (ssGSEA) was used to clarify the enrichment information of immune function-related genes in two different risk groups by the R package “GSEAbase.” Then, R-package “ESTIMATE” was utilized to evaluate the purity of tumor, the degree, and level of immune cells and stromal cells. The CIBERSORT package was used to analyze the immune infiltration of each sample.

### 2.9. Relationship between Risk Score and Immune Checkpoint

To clarify the potential role of RMA-AS_Score signature in the treatment of immune checkpoint in HNSCC, we correlated the risk score and six immune checkpoint key genes. Finally, the expression levels of 35 immune checkpoint genes in two different risk groups were compared.

### 2.10. Construction of Splicing Regulatory Network

First, we download the RNA-seq profiles of splicing factors from TCGA-HNSCC database. Spearman correlation analysis was employed to assess the correlation between splicing factors and RMA-AS events. Set *P* < 0.05 and correlation coefficient > 0.6 as a threshold. Finally, a regulatory network of splicing factor-regulated RMA-AS was constructed using Cytoscape (version 3.8.0).

## 3. Results

### 3.1. Identification of AS Events in HNSCC

**Figure 2:**
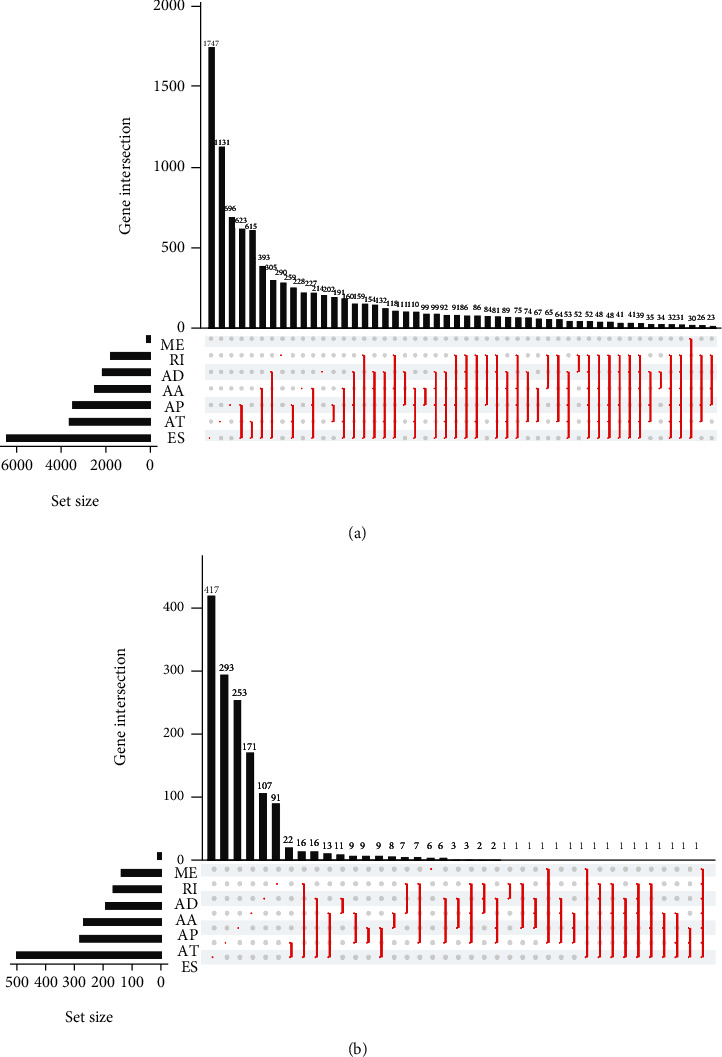
AS events profiling in HNSCC. (a) UpSet plot of all AS events in HNSCC. (b) UpSet plot of prognosis-related AS events in HNSCC.

We downloaded AS data of HNSCC from TCGA SpliceSeq and obtained 42,849 mRNA AS events (Table S1). By comprehensively analyzing the profiles of AS events, the gene crossover among the seven types of AS events was shown in the UpSet diagram. Among all these AS events of HNSCC, ES was the most splicing pattern, while ME was the rarest (Figure 2(a)). Subsequently, 16,574 AS events related with prognosis were obtained by univariate Cox regression analysis. We exhibited the UpSet plot of prognostic-related AS events, and the results showed ES was the predominant prognosis-associated AS event, and ME was the least (Figure 2(b)).

### 3.2. Analysis of AS Events Associated with RNA Modification

**Figure 3:**
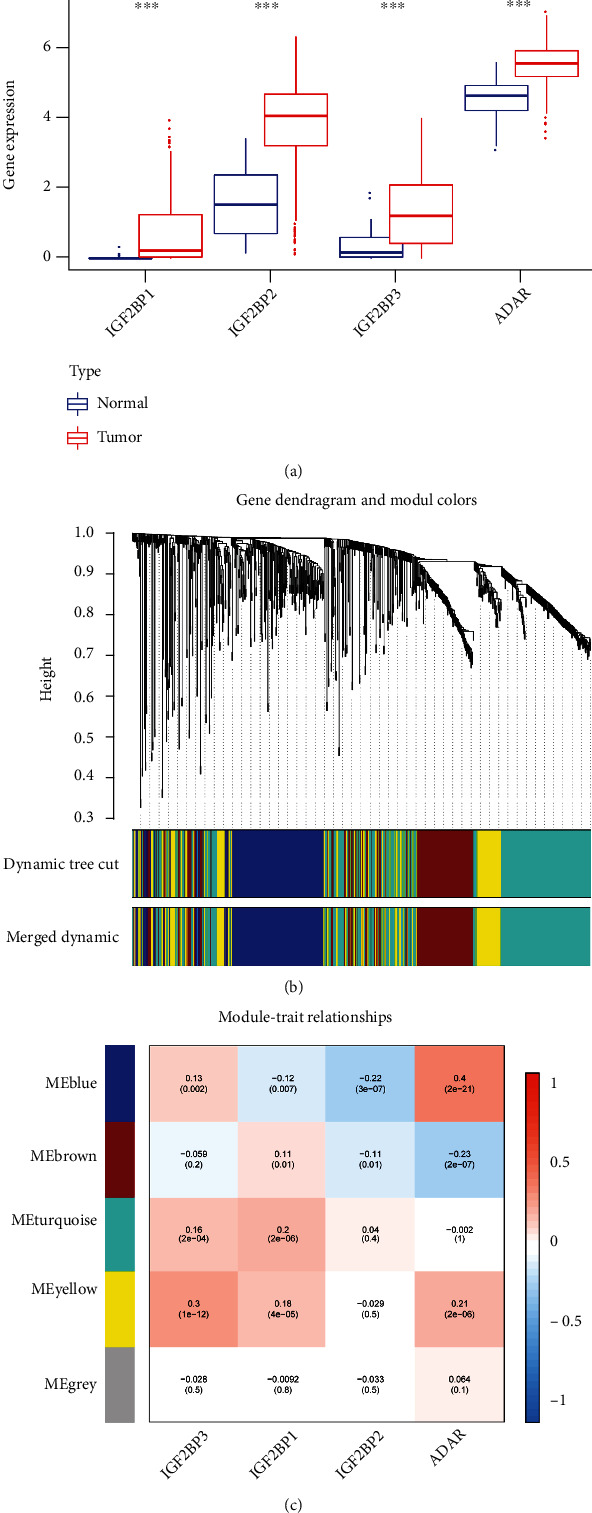
Identification of the prognosis-related AS events that are associated with the expression of RNA-modified genes. (a) Expression of RNA-modified genes in cancer and normal tissues. (b) Hierarchical clustering tree of the RMA-AS events in TCGA-HNSCC. (c) Hierarchical clustering tree of the RMA-AS events in TCGA-HNSCC. (d) Association of the AS events with RNA modified genes.

Firstly, the expression information of 55 genes related to RNA modification was extracted from the TCGA-HNSCC database (Table S2). A total of 4 differentially expressed genes, including IGF2BP1, IGF2BP2, IGF2BP3, and ADAR, were identified between HNSCC tissues and normal tissues (Figure 3(a)). Subsequently, to obtain RMA-AS events, we constructed a coexpression network between these 4 differentially expressed genes related to RNA modification and the above prognosis-associated AS events, by WGCNA package (Figure 3(b)). A *P* value < 0.05 was considered that there was a significant correlation between genes and AS events. We found that 4 modules (including blue, brown, turquoise, and yellow modules) of AS events were significantly coexpressed with the 4 RNA modification-related genes (Figure 3(c)). For example, the AS events of brown module were negatively correlated with IGF2BP2 and ADAR, while positively correlated with IGF2BP1. Additionally, AS events of blue module were positively correlated with IGF2BP3 and ADAR and negatively correlated with IGF2BP1 and IGF2BP2. Therefore, the AS events of the four modules that coexpressed with modification-related genes, also defined as RMA-AS events, were used for subsequent analysis.

### 3.3. Establishment of the Prognostic Risk Score Model of RMA-AS Events

**Figure 4:**
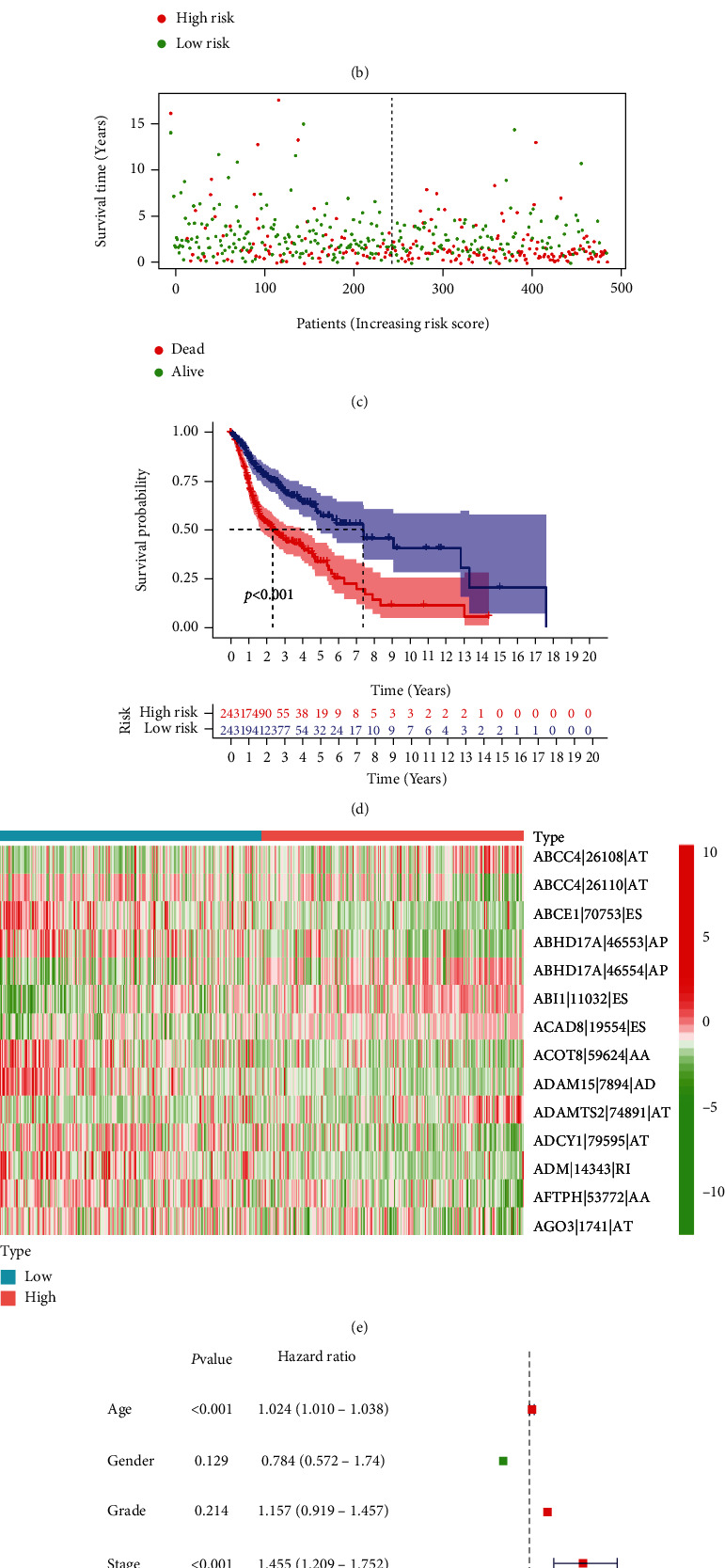
Confirmation of RMA-AS_Score prognostic signature. (a) LASSO regression analysis was performed for the RMA-AS events. (b) Distribution of risk score in RMA-AS_Score signature. (c) The survival status of HNSCC patients. (d) The results of Kaplan-Meier curve. (e) Heatmap of RMA-AS event PSI value. (f) The results of univariate Cox regression analysis. (g) The results of multivariate Cox regression analysis.

Based on the above RMA-AS events, we constructed a risk score model for HNSCC (this model termed as RMA-AS_Score). We first obtained the RMA-AS_Score composed of 14 AS events by LASSO Cox regression analysis (Figure 4(a)). Then, according to the risk score, the samples were divided into RMA-AS_Score high- and RMA-AS_Score low-risk groups. As shown in Figures 4(b) and 4(c), the allocations of risk score and dot pot of survival status revealed that the patients of the RMA-AS_Score high-risk group have a shorter OS. Similarly, Kaplan-Meier curve confirmed that patients of RMA-AS_Score-high have poorer prognosis than patients in the RMA-AS_Score low group (Figure 4(d)). We also drew a heatmap to display the expression profiles of these 14 AS events (Figure 4(e)). We found that patients with RMA-AS_Score-low tended to express high levels of protective AS events (ABCC4-26110-AT, ABCE1-70753-ES, ABHD17A-46553-AP, ACTO8-19554-ES, ADAM15-7894-AD, ADCY1-79595-AT, ADM-14343-RI, AFTPH-53772-AA, and AGO3-1741-AT), whereas patients with RMA-AS_Score-high exhibited a preference for high levels of the other 5 AS events (ABCC4-26108-AT, ABHD17A-46554-AP, ABI1-11032-ES, ADAD8-19554-ES, and ADAMTS2-74891-AT). Moreover, univariate Cox analysis revealed that the hazard ratio (HR) of risk score was 1.070 (95% confidence interval (CI): 1.043−1.099; Figure 4(f)). Multivariate Cox analysis further showed that risk score (HR = 1.072, 95% CI: 1.044−1.100; Figure 4(g)) was an independent prognostic factor in HNSCC. Therefore, RMA-AS events can be used as a prognostic risk model for HNSCC.

### 3.4. Correlation of Prognostic Signature with Clinical Features and Construction of RMA-AS-Clinicopathological Nomogram

**Figure 5:**
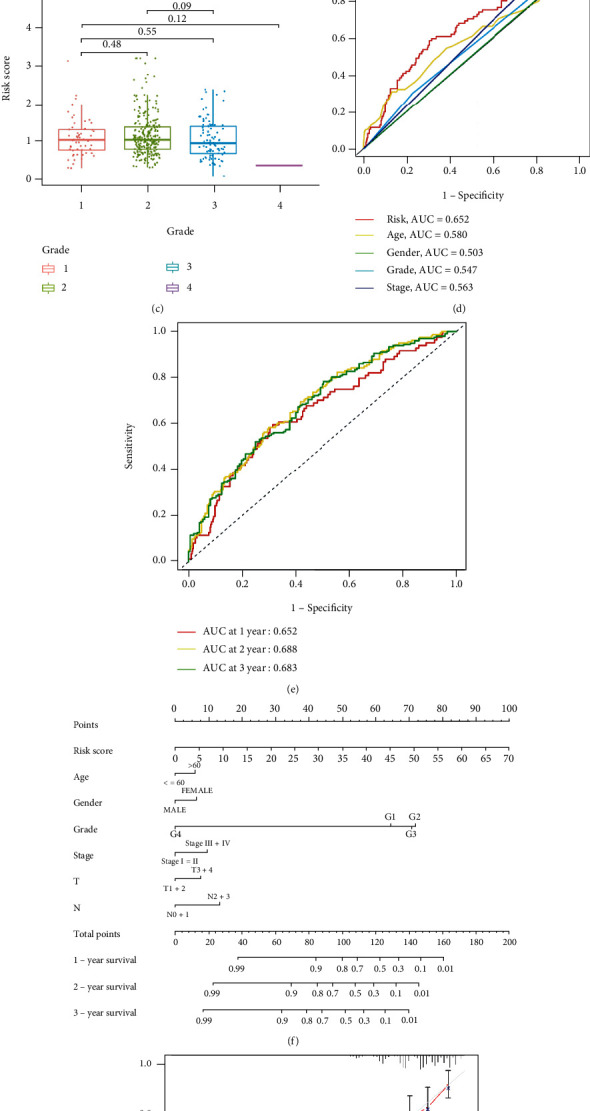
Correlation between RMA-AS_Score prognostic signature and clinical features and construction of the clinicopathological map. (a–c) Correlation of risk score with gender, stage, and tumor grade. (d) The AUC of RMA-AS_Score prognostic signature and clinical parameters. (e) The AUC of 1-, 2-, and 3-year survival. (f) Nomogram was constructed by using RMA-AS_Score signature and clinical parameters. (g) The nomogram calibration plot in HNSCC.

We previously used univariate and multivariate regression analyses to evaluate whether our signature is independent of clinical indicators, which confirmed that the RMA-AS_Score model is an independent prognostic factor. Therefore, we further analyzed clinical significance of the RMA-AS_Score. In addition to gender and clinicopathological stage, the RMA-AS_Score increased markedly with advanced tumor grade (Figures 5(a)–5(c)). To explore whether the RMA-AS_Score was the best prognostic index, we selected age, gender, clinicopathological stage, and tumor grade as candidate predictors of prognosis. As shown in Figure 5(d), the RMA-AS_Score possessed the largest AUC value (0.652). Furthermore, 1-, 2-, and 3-year AUC of the RMA-AS_Score model was 0.652, 0.688, and 0.683, respectively (Figure 5(e)). Finally, we constructed a nomogram plot of the RMA-AS_Score (Figure 5(f)) and the calibration curve (Figure 5(g)) to evaluate the accuracy of the nomogram plot, suggesting our nomogram plot had wonderful predictive power. In summary, the RMA-AS_Score can significantly improve the prognostic ability of HNSCC.

### 3.5. Correlation between RMA-AS_Score and Immune Microenvironment Characterization

**Figure 6:**
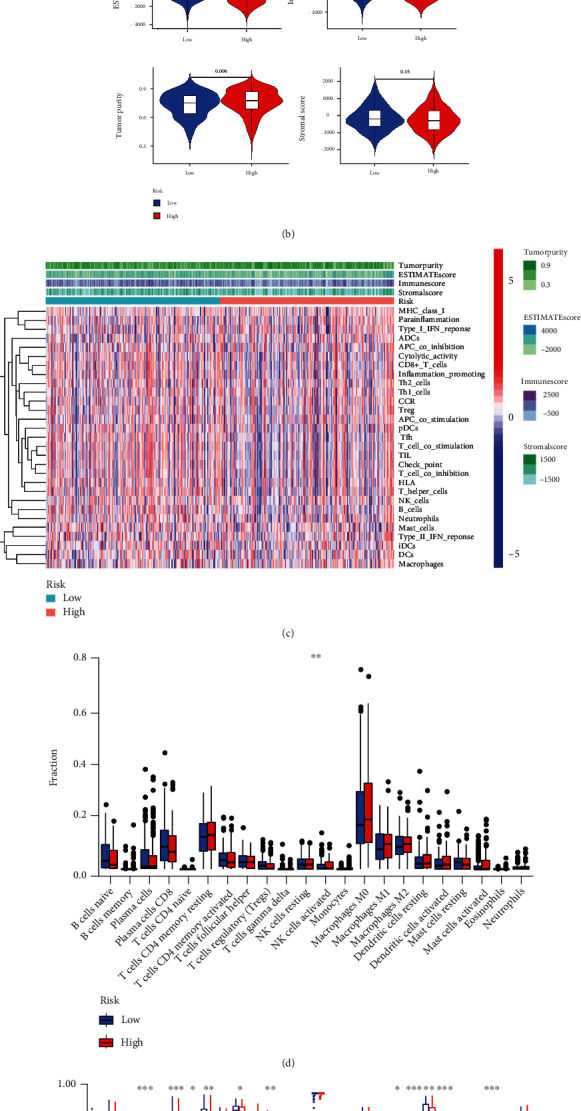
Association between infiltrating immune cells and RMA-AS_Score prognostic signature. (a) Relationship between the RMA-AS_Score signature and immune cells. (b) The assessment of the purity of tumor, the degree, and level of immune cells and stromal cells. (c) Heatmap of immune signatures and immune scores of two different risk groups. (d) Expression levels of immune cell in two different risk groups. (e) Enrichment information of immune-related signatures in two different risk groups.

Exploring the relationship between the prognosis model and tumor immune microenvironment can provide therapeutic benefits for the development of immunotherapy and may contribute to the clinical decision-making of cancer patients. Hence, we performed the correlation analysis for prognostic risk score and tumor immune environment characterization to confirm whether RMA-AS_Score can act as an immune indicator [12]. Firstly, the RMA-AS_Score signature showed the evident negative association with T cell regulatory (Treg), T cell follicular helper, and B cell naïve, while positive association with macrophage M2 (Figure 6(a)), indicating the RMA-AS_Score signature is related to the HNSCC immune microenvironment. Likewise, RMA-AS_Score low patients obtained higher estimate score and immune score, lower tumor purity which represented more immune infiltration (Figure 6(b)).  Figure 6(c) exhibits the immune-related signatures of each patient in the RMA-AS_Score high-/low-risk groups, which suggested patients with a low risk score were rich in stromal and immune cells and had fewer pure tumors. Subsequently, we found the infiltration of Treg expression level was observably decreased with advanced risk score (Figure 6(d)). The CIBERSORT algorithm results suggested that the proportion of B cells, CD8+ T cells, checkpoint, cytolytic activity, HLA, inflammation promoting, T cell coinhibition/simulation, T helper cells, Tfh, and TIL was also declined with growth risk score (Figure 6(e)). Combined with the above results, different risk groups of HNSCC can accurately indicate the immune level, and the overall immune response level of patients in the high-risk group is lower than that in the low-risk group.

### 3.6. Correlation between RMA-AS_Score Signature and Immune Checkpoint Key Molecules

**Figure 7:**
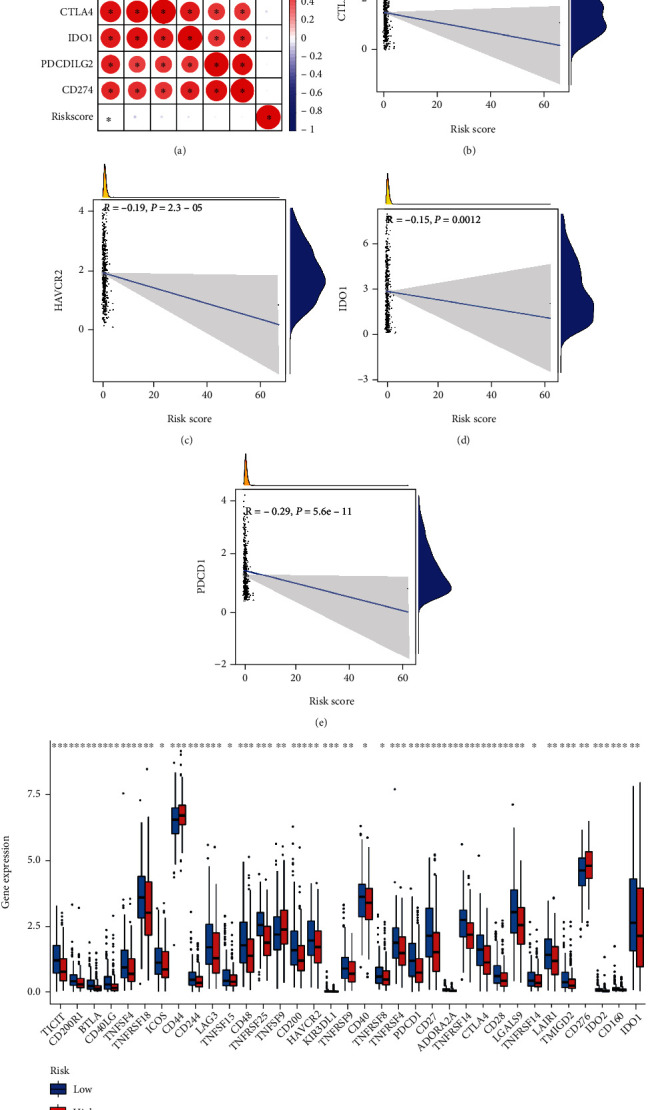
Correlation between RMA-AS_Score prognostic signature and immune checkpoint. (a) Correlation association analyses between risk score and immune checkpoint. (b–e) Correlation between risk score and immune checkpoint. (f) Comparison of immune checkpoint-related gene expression levels among different risk groups.

To further investigate the effect of our prognostic model on the immune system, we analyzed the relationship between immune checkpoint and RMA-AS_Score signature. Firstly, the correlation between immune checkpoint key molecules and RMA-AS_Score prognostic indicators was analyzed to reveal potential participants of risk indicators in the immune detection point treatment of HNSCC (Figure 7(a)). The results hinted that RMA-AS_Score signature was notably negative related to CTLA4, HAVCR2, IDO1, and PDCD1 (Figures 7(b)–7(e)). Further correlation analysis showed that compared with the RMA-AS_Score low group, the expression levels of CD44, TNFSF9, and CD276 were upregulated in patients in the RMA-AS_Score high group, while the others were almost downregulated (Figure 7(f)). Taken together, in HNSCC immunotherapy, we need to pay attention to the adverse factors in RMA-AS_Score prognostic signature.

### 3.7. Correlation Analysis of RMA-AS and Splicing Factors

**Figure 8:**
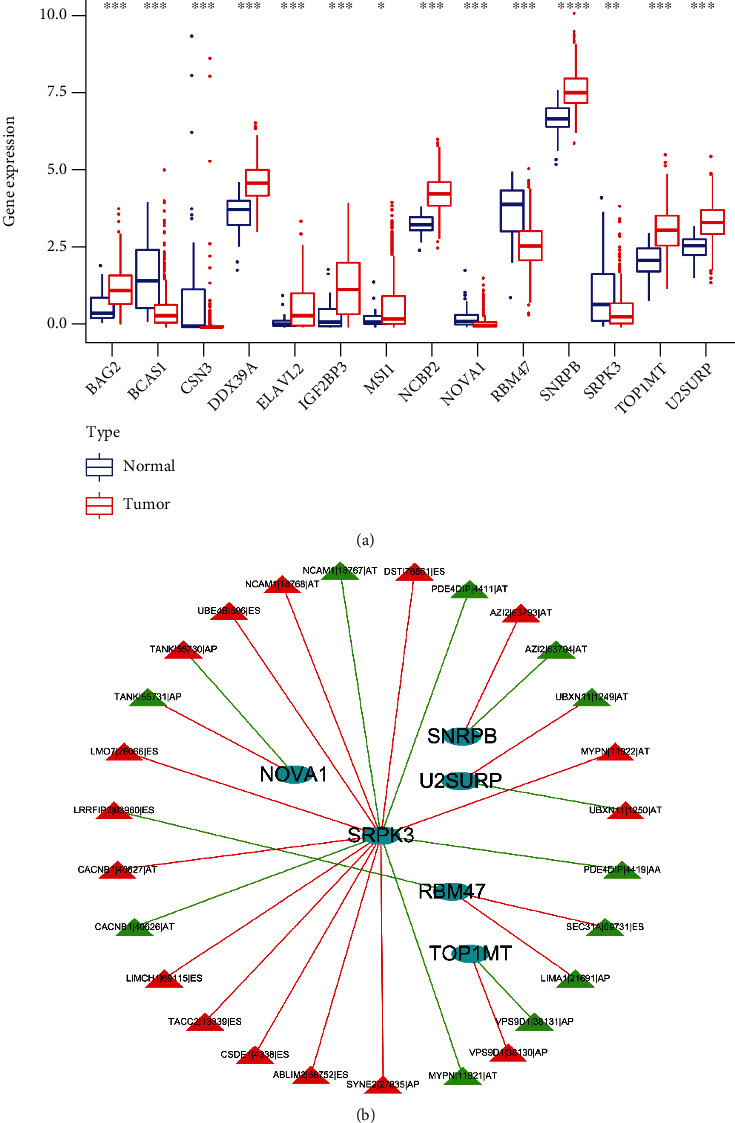
Analysis of the relationship between RMA-AS and splicing factors. (a) Expression of splicing factors in cancer and normal tissues. (b) The regulatory network of splicing factor-regulated RMA-AS. Oval represents splicing factors, the triangle represents RMA-AS, the red represents high-risk AS, and the green represents low-risk AS.

To screen for splicing factor-regulated RMA-AS, we analyzed differentially expressed splicing factors in tumor and normal tissues. The results demonstrated a total of 14 differentially expressed splicing factors were obtained (Figure 8(a)). In addition, we also constructed a correlation network of splicing factor-regulated RMA-AS (Figure S1). In our regulatory network, we also found the six most important splicing factors, including NOVA1, SNRPB, U2SURP, SRPK3, TOP1MT, and RBM47 (Figure 8(b)). SRPK3, in particular, regulated 11 high-risk and 5 low-risk AS events. Therefore, these splicing factors indicated a great potential to act as central regulators involving in the dysregulation of RMA-AS in HNSCC.

## 4. Discussion

HNSCC is a common heterogeneous tumor with high mortality and poor prognosis [13]. Therefore, it is urgent to find a tool to predict HNSCC prognosis, which contributes the development of individualized treatment of HNSCC. Increasing studies support that RNA modification and AS events play a role in physiological and pathological processes [8, 14]. However, little is known about the correlation between RMA-AS events and HNSCC prognosis.

In this study, we found 42,849 AS events in HNSCC from TCGA SpliceSeq database and obtained 16,574 AS associated with prognosis and 4 differential RNA modification-related genes. Subsequently, RMA-AS events were acquired by constructing AS events coexpressed with differentially expressed RNA modification-related genes. According to the RMA-AS events, we constructed a risk model composed of 14 AS events, termed as RMA-AS_Score. Based on prognostic signatures of RMA-AS_Score, HNSCC patients with high-risk had a shorter OS. Besides, the RMA-AS_Score was an independent indicator in HNSCC by univariate and multivariate response analyses. We also built a nomogram to certify that the prediction model is consistent with the actual results. By analyzing the immune microenvironment, we found that the RMA-AS_Score low group had stronger antitumor ability compared with the RMA-AS_Score high group. Finally, we identified 6 splicing factors, which may be involved in the dysregulation of RMA-AS in HNSCC as central regulatory factors. Collectively, our prognostic model provides reliability for improving the prognosis of HNSCC.

The abnormal AS patterns found in cancer can help identify disorders in cell function. Some studies have reported that the splicing changes of genes during tumorigenesis directly affect the pathway of apoptosis and proliferation, resulting in regulating the development of tumors [15]. In addition, it is of great value to predict the prognosis of cancer by establishing the prediction model of AS events. Xu et al. have found that 3,294 AS events were associated with survival in hepatocellular carcinoma [16]. Similarly, we screened 16,574 AS associated with prognosis in HNSCC, which provides a basis for the later construction of prognosis model.

With the rapid development of RNA research, it is gradually found that the imbalance of RNA modification is closely related to the pathological processes related to carcinogenesis [17]. Zhang et al. reported that YTHDF2, an m6A reader, is abnormally expressed in hepatocellular carcinoma, which promotes tumor metastasis by regulating the methylation of OCT4 mRNA m6A [18]. Furthermore, m6A-related regulatory factors were abnormally expressed in osteosarcoma, and HNRNPA2B1 may be an independent risk factor for OS [19]. In our study, we found 4 differential expression genes of RNA modification in HNSCC, indicating that these genes are involved in HNSCC progression. Combined with the signature of RMA-AS events, which can be used to predict the prognosis of tumors [20], we also established an RMA-AS risk model of HNSCC, named RMA-AS_Score. In the RMA-AS_Score, we divided patients into high- and low-risk according to the risk score, among which the OS of high-risk patients was obviously shorter than that of low-risk patients. Moreover, we selected ABCC4, ABCE1, ABHD17A, ABI1, ACAD8, ACTO8, ADAM15, ADAMTS2, ADCY1, ADM, AFTPH, and AGO3 as RMA-AS event-associated signatures for predictive prognosis in HNSCC. Some literature have been reported reducing the expression of ABBC4 in pancreatic cancer cells significantly inhibited the proliferation and migration of cancer cells [21]. AFTPH (Aftiphilin) was highly expressed in diffuse large B cell lymphoma, pancreatic adenocarcinoma, breast cancer, and lung squamous cell carcinoma and linked to poor prognosis [22]. Besides, high expression of ADAM15 was an independent risk factor for the prognosis of hepatocellular carcinoma [23]. Similarly with other risk models [24], we also determined that the prognostic risk model is an independent clinical index through univariate and multivariate response analyses, indicating that the RMA-AS_Score is an independent prognostic factor. More importantly, the AUC of our risk model was superior to other clinical indicators, which reflects the application value of RMA-AS_Score in HNSCC. In this regard, our RMA-AS_Score provided a guarantee for predicting the prognosis of HNSCC, and the 14-RNA modification-related AS events could act as potential prognostic and therapeutic targets of HNSCC.

To reveal the role of RMA-AS events in the immune microenvironment in HNSCC, we also performed ESTIMATE algorithm, ssGSEA method, and CIBERSORT analysis. Similar to other reports [25], our estimated score and immune score in the low-risk group were higher than those in the high-risk group, suggesting the higher the number of immune cells in the low-risk group, the lower the number of tumor cells, and the stronger the immunity. In addition, we found that M2 macrophages were negatively correlated with a risk score. Since M2 macrophages are mainly involved in the anti-inflammatory response [26], this explains why the anti-immune ability is weakened in the high-risk group. Furthermore, immune checkpoint detection proved that the expression level of immune checkpoint was low in the high-risk group [27]. Given that the immunotherapy against immune checkpoints in HNSCC has not achieved satisfactory results, we speculate that this may be related to the low expression level of immune checkpoints in our RMA-AS_Score high patients. In this case, our RMA-AS_Score emphasized the value in tumor immune response of HNSCC, and targeting immune detection proteins could guide HNCC treatment.

The change of AS may originate from the change of splicing factor expression, which affects the splicing of cancer-related genes [28]. Splicing factors can be used as tumor proteins or tumor suppressors and drug targets for tumor therapy [29], among which the disorder of its level will affect the pathogenesis of cancer [30]. In view of this, it is important to find the regulatory network between RMA-AS and related splicing factors. In this study, we constructed a network of splicing factor-regulated RMA-AS and found several important splicing factors, such as NOVA1, SNRPB, U2SURP, SRPK3, TOP1MT, and RBM47. Although these genes regulate various AS events, their potential role in the pathogenesis and development of HNCSS remains to be studied.

## 5. Conclusion

We constructed RMA-AS_Score prognostic model to strengthen the prognostic prediction of HNSCC. The 14-RMA-AS signature demonstrated great potential of predictive ability and independent from clinical characteristics. In addition, we further analyzed the value of RMA-AS_Score model in the immune microenvironment, providing a basis for the development of personalized immunotherapy for HNSCC. Besides, the regulatory network of splicing factor-regulated RMA-AS indicated promising targets of the antitumor therapy in HNSCC. Collectively, we established a robust method to deeply study the etiology and progress of HNSCC, which also provides a reference for the research of other cancers.

## Data Availability

Data are available on request.

## Supplementary Materials

Supplementary 1 Figure S1: the regulatory network of all splicing factor-regulated RMA-AS. Oval represents splicing factors, triangle represents RMA-AS, red represents high-risk AS, and green represents low-risk AS.

Supplementary 2 Table S1: details of alternative splicing data. Table S2: list of genes related to RNA modifications in TCGA database.
