# Supplementary figures and images for: Identification of RNA Modification-Associated Alternative Splicing Signature as an Independent Factor in Head and Neck Squamous Cell Carcinoma
Source: J Immunol Res. 2022 Sep 13;2022:8976179. doi: 10.1155/2022/8976179 (PMC9490063; doi:10.1155/2022/8976179)

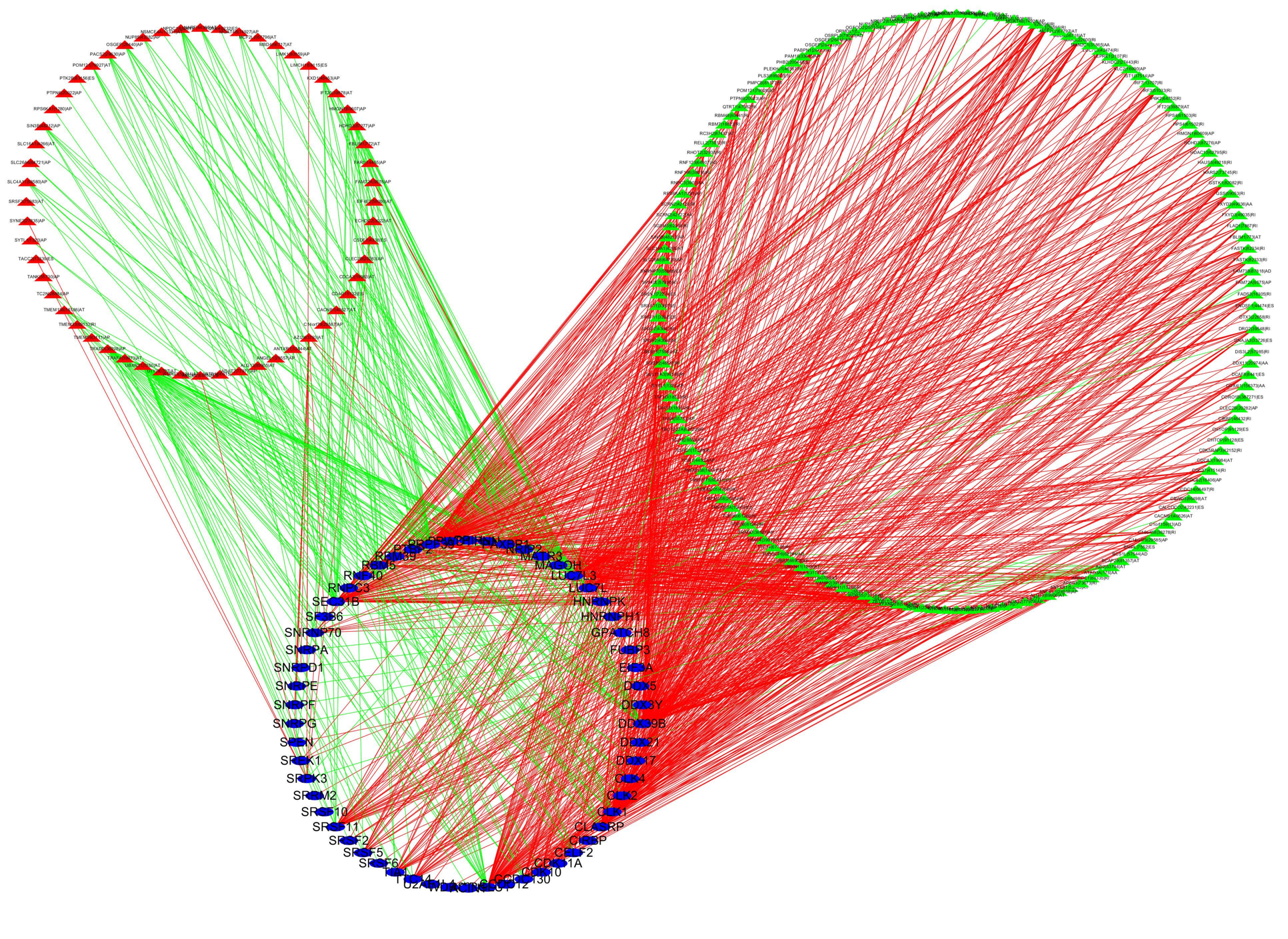

Supplement: Supplementary 1 — Figure S1: the regulatory network of all splicing factor-regulated RMA-AS. Oval represents splicing factors, triangle represents RMA-AS, red represents high-risk AS, and green represents low-risk AS. [file 8976179.f1.pdf]
