# Supplementary material for: Identification of RNA Modification-Associated Alternative Splicing Signature as an Independent Factor in Head and Neck Squamous Cell Carcinoma
Source: J Immunol Res. 2022 Sep 13;2022:8976179. doi: 10.1155/2022/8976179 (PMC9490063; doi:10.1155/2022/8976179)
Supplement: Supplementary 2 — Table S1: details of alternative splicing data. Table S2: list of genes related to RNA modifications in TCGA database. [file 8976179.f2.doc]

**Table S2** List of genes related to RNA modifications in TCGA database

| Genes |
| --- |
| METTL3 |
| METTL14 |
| WTAP |
| RBM15 |
| RBM15B |
| ZC3H13 |
| VIRMA(KIAA1429) |
| METTL16 |
| FTO |
| ALKBH5 |
| IGF2BP1 |
| IGF2BP2 |
| IGF2BP3 |
| YTHDF1 |
| YTHDF2 |
| YTHDF3 |
| YTHDC1 |
| YTHDC2 |
| HNRNPC |
| HNRNPG |
| eIF3 |
| ELAVL |
| HNRNPA2B1 |
| RBMX |
| HuR |
| FMR1 |
| SRSF2 |
| LRPPRC |
| TRMT61A |
| TRMT10C |
| TRMT61B |
| TRMT6 |
| NML |
| ALKBH1 |
| ALKBH3 |
| CPSF1 |
| CPSF2 |
| CPSF3 |
| CPSF4 |
| CPSF6 |
| CPSF7 |
| CSTF1 |
| CSTF2 |
| CSTF3 |
| CFI |
| PCF11 |
| CLP1 |
| WDR33 |
| FIP1L1 |
| PABPN1 |
| RBBP6 |
| NUDT21 |
| ADAR |
| ADARB1 |
| ADARB2 |
